# Erratum

**Published:** 2015-08-14

## Vol. 64, No. 30

In the report, “School Start Times for Middle School and High School Students — United States, 2011–12 School Year,” an error occurred in the arrangement of column headings in the table on pages 811 and 812. The corrected table follows.

## Vol. 64, No. 30

In the report, “Lack of Measles Transmission to Susceptible Contacts from a Health Care Worker with Probable Secondary Vaccine Failure — Maricopa County, Arizona, 2015,” on page 833, the last sentence of the report, “2 doses of MMR vaccine, administered ≥28 days apart, are recommended for children aged ≥12 months and adults born after 1956, for prevention of measles,” should be replaced with the sentence: **“All vaccine-eligible persons aged ≥12 months should receive the age-appropriate number of MMR vaccine doses unless they have other evidence of measles immunity.”**

## Figures and Tables

**TABLE:** Average start time and percentage distribution of start times for public middle, high, and combined schools,* by school level and state — Schools and Staffing Survey 2011–12 school year

School level and state	Estimated no. of public middle, high, and combined schools		Estimated no. of students in public middle, high, and combined schools		Average start time (a.m.)¶		Percentage distribution† of public middle, high, and combined school start times							

							Before 7:30 a.m.		7:30 a.m. to 7:59 a.m.		8:00 a.m. to 8:29 a.m.		8:30 a.m. or later	
						
	No.	(SE)	No.	(SE)	Time	(SE)§	%	(SE)	%	(SE)	%	(SE)	%	(SE)
**Total**	**39,700**	**(390)**	**26,284,000**	**(613,100)**	**8:03**	**(1)**	**6.7**	**(0.4)**	**31.9**	**(0.8)**	**43.7**	**(0.8)**	**17.7**	**(0.7)**
**School level**														
Middle	13,990	(169)	8,674,000	(135,800)	8:04	(1)	4.8	(0.7)	35.9	(1.3)	40.4	(1.1)	18.9	(1.0)
High	18,360	(434)	14,995,000	(413,600)	7:59	(1)	9.5	(0.6)	33.0	(1.1)	43.1	(1.1)	14.4	(0.9)
Combined	7,350	(571)	2,615,000	(300,600)	8:08	(3)	3.5	(0.7)	21.6	(2.2)	51.5	(2.6)	23.4	(2.7)
**State**														
Alabama	680	(39)	344,000	(31,100)	7:49	(2)	6.4	(2.2)††	57.8	(4.4)	34.0	(5.3)	—**	—
Alaska	—**	—	—**	—	8:33	(8)	0.0	—§§	11.6	(3.8)††	11.6	(4.8)††	76.8	(7.8)
Arizona	860	(159)	506,000	(53,100)	8:03	(3)	8.1	(2.9)††	23.3	(6.6)	47.3	(5.8)	21.3	(5.0)
Arkansas	450	(28)	292,000	(30,300)	8:01	(1)	—**	—	29	(4.7)	63.0	(4.7)	7.3	(2.0)
California	3,880	(219)	3,303,000	(146,300)	8:07	(2)	3.5	(0.9)	27.7	(3.1)	47.6	(3.3)	21.2	(2.9)
Colorado	730	(84)	527,000	(51,700)	7:54	(2)	16.9	(5.1)	31.3	(6.6)	40.9	(5.1)	10.9	(2.6)
Connecticut	380	(24)	260,000	(23,900)	7:46	(2)	13.8	(2.9)	57.4	(4.2)	24.0	(3.8)	4.8	(2.1)††
Delaware	090	(4)	63,000	(4,900)	7:42	(3)	24.0	(5.3)	51.9	(6.3)	16.6	(4.6)	7.5	(3.0)††
District of Columbia	—**	—	—**	—	—**	—	—**	—	—**	—	—**	—	—**	—
Florida	1,570	(100)	1,406,000	(111,400)	8:17	(3)	19.5	(2.5)	18.6	(2.4)	19.3	(2.9)	42.6	(3.8)
Georgia	1,030	(24)	955,000	(77,500)	8:09	(2)	—**	—	28.7	(4.3)	43.9	(4.6)	24.0	(3.4)
Hawaii	—**	—	—**	—	8:03	(3)	0.0	—§§	42.5	(17.3)††	57.5	(17.3)††	0.0	—§§
Idaho	370	(182)	157,000	(40,300)	8:13	(28)	0.0	—§§	20.9	(7.5)††	58.3	(14.5)	—**	—
Illinois	1,590	(48)	1,008,000	(145,200)	8:13	(3)	—**	—	19.7	(3.4)	48.7	(5.5)	28.4	(6.0)
Indiana	740	(27)	559,000	(43,800)	7:58	(2)	—**	—	41.8	(3.2)	45.1	(4.0)	10.2	(2.7)
Iowa	550	(35)	249,000	(31,300)	8:23	(6)	0.0	—§§	6.3	(2.0)††	66.3	(7.2)	27.4	(7.6)
Kansas	540	(20)	204,000	(20,000)	8:00	(1)	—**	—	26.5	(3.5)	71.5	(3.7)	—**	—
Kentucky	710	(32)	358,000	(33,100)	8:03	(4)	8.6	(4.2)††	24.8	(4.0)	49.0	(5.8)	17.5	(4.0)
Louisiana	630	(32)	316,000	(33,100)	7:40	(2)	29.9	(4.8)	53.1	(4.9)	12.1	(3.5)	—**	—
Maine	240	(5)	105,000	(5,500)	7:53	(3)	6.6	(1.9)	53.1	(5.1)	32.8	(4.8)	7.5	(3.6)††
Maryland	—**	—	—**	—	—**	—	—**	—	—**	—	—**	—	—**	—
Massachusetts	700	(58)	527,000	(48,600)	7:53	(4)	8.0	(3.6)††	53.3	(6.1)	27.2	(5.1)	11.5	(5.4)††
Michigan	1,540	(47)	891,000	(59,100)	7:54	(2)	9.5	(2.1)	43.6	(3.6)	39.0	(3.5)	7.9	(2.2)
Minnesota	1,100	(58)	522,000	(43,100)	8:18	(3)	0.9	(0.4)††	18.8	(2.6)	46.7	(3.7)	33.6	(3.5)
Mississippi	570	(23)	272,000	(18,600)	7:47	(2)	12.4	(3.7)††	58.3	(4.3)	29.3	(4.3)	0.0	—§§
Missouri	900	(37)	530,000	(28,700)	7:54	(1)	6.7	(1.7)	39.0	(3.9)	51.0	(3.9)	3.2	(1.4)††
Montana	220	(15)	78,000	(8,200)	8:13	(2)	0.0	—§§	5.8	(2.1)††	80.9	(6.1)	13.4	(5.5)††
Nebraska	370	(26)	150,000	(19,200)	8:07	(1)	0.0	—§§	8.0	(2.5)††	88.9	(2.4)	3.0	(1.4)††
Nevada	260	(12)	276,000	(20,900)	7:51	(3)	18.0	(3.0)	30.7	(5.5)	38.2	(6.0)	13.1	(3.6)
New Hampshire	180	(18)	116,000	(7,800)	7:46	(2)	11.6	(3.2)	64.4	(5.7)	19.7	(4.4)	—**	—
New Jersey	870	(52)	698,000	(45,200)	8:00	(2)	6.7	(2.0)	37.2	(4.5)	41.2	(4.7)	14.9	(3.6)
New Mexico	310	(99)	151,000	(47,000)	8:10	(3)	1.6	(0.7)††	24.1	(5.8)	53.9	(10.2)	20.4	(5.9)
New York	2,070	(108)	1,670,000	(149,100)	7:59	(2)	7.7	(3.1)††	31.6	(2.9)	49.6	(3.4)	11.0	(2.5)
North Carolina	1,120	(35)	768,000	(88,900)	8:03	(2)	—**	—	36.6	(5.0)	45.3	(5.4)	15.2	(4.2)
North Dakota	220	(9)	67,000	(5,000)	8:31	(1)	0.0	—§§	2.8	(1.2)††	18.7	(3.2)	78.5	(3.4)
Ohio	1,640	(73)	1,061,000	(60,800)	7:52	(2)	13.1	(2.0)	45.3	(4.3)	29.3	(3.7)	12.3	(3.0)
Oklahoma	700	(27)	356,000	(29,000)	8:10	(2)	0.0	—§§	12.0	(2.8)	77.6	(3.9)	10.4	(2.8)
Oregon	480	(25)	282,000	(21,100)	8:14	(3)	—**	—	25.2	(3.8)	45.0	(4.1)	28.9	(4.2)
Pennsylvania	1,280	(145)	1,001,000	(189,700)	7:48	(2)	13.0	(3.0)	51.3	(6.6)	32.6	(7.9)	3.1	(1.3)††
Rhode Island	100	(10)	68,000	(6,200)	7:50	(4)	24.8	(6.1)	27.5	(7.9)	40.3	(9.2)	—**	—
South Carolina	500	(9)	411,000	(26,400)	8:03	(2)	—**	—	35.3	(6.5)	50.9	(6.8)	12.3	(3.7)
South Dakota	230	(11)	78,000	(5,200)	8:13	(2)	—**	—	6.6	(2.7)††	77.7	(4.2)	14.8	(4.9)††
Tennessee	760	(47)	533,000	(31,000)	7:57	(3)	13.3	(3.4)	29.4	(4.7)	40.0	(5.1)	17.2	(3.5)
Texas	3,940	(183)	2,556,000	(254,700)	8:05	(2)	3.1	(1.2)††	28.3	(3.4)	46.3	(3.5)	22.4	(2.7)
Utah	410	(22)	297,000	(45,200)	8:05	(3)	0.0	—§§	33.1	(5.3)	49.6	(5.9)	17.3	(5.9)††
Vermont	100	(2)	46,000	(2,600)	8:05	(2)	—**	—	34.1	(5.1)	48.0	(4.8)	15.1	(3.0)
Virginia	850	(17)	555,000	(37,700)	8:04	(2)	10.0	(2.6)	26.6	(4.4)	42.6	(4.4)	20.8	(3.6)
Washington	930	(35)	526,000	(42,300)	8:08	(2)	6.4	(1.9)††	24.2	(3.8)	50.2	(4.6)	19.3	(3.5)
West Virginia	300	(5)	160,000	(7,000)	7:54	(2)	11.1	(2.0)	33.9	(3.3)	47.9	(4.0)	7.1	(2.3)††
Wisconsin	860	(37)	423,000	(44,200)	7:59	(3)	2.3	(1.0)††	48.2	(5.4)	39.1	(4.3)	10.4	(4.4)††
Wyoming	130	(8)	50,000	(4,300)	7:59	(1)	0.0	—§§	41.1	(5.2)	58.9	(5.2)	0.0	—§§

**Source:** U.S. Department of Education, National Center for Education Statistics, Schools and Staffing Survey (SASS), “Public School Data File,” 2011–12.

**Abbreviation:** SE = standard error.

* Middle schools include any schools with no grade lower than 5 and no grade higher than 8. High schools include any school with no grade lower than 7 and at least one grade higher than 8. Combined schools include any schools with at least one grade lower than 7 and at least one grade higher than 8, or with all students in ungraded classrooms.

† Detail may not sum to totals because of rounding and because some data are not shown.

§ SE of average start time is expressed in minutes.

¶ Schools with afternoon start times were not included in analysis.

** Reporting standards not met. Relative standard error =0.5 or the response rate <50%.

†† Interpret data with caution. 0.3 = relative standard error < 0.5.

§§ Rounds to zero. SE is not applicable.

